# Epidemiological evidence relating environmental smoke to COPD in lifelong non-smokers: a systematic review

**DOI:** 10.12688/f1000research.13887.3

**Published:** 2020-01-09

**Authors:** Peter N. Lee, Barbara A. Forey, Katharine J. Coombs, Jan S. Hamling, Alison J. Thornton

**Affiliations:** 1 P.N. Lee Statistics and Computing Ltd, Sutton, Surrey, SM2 5DA, UK; 2 Independent Consultant in Statistics, Okehampton, Devon, EX20 1SG, UK

**Keywords:** Passive smoking, COPD, Dose-response, Meta-Analysis, Review, Pulmonary Disease

## Abstract

**Background: **Some evidence suggests environmental tobacco smoke (ETS) might cause chronic obstructive pulmonary disease (COPD). We reviewed available epidemiological data in never smokers.

**Methods: **We identified epidemiological studies providing estimates of relative risk (RR) with 95% confidence interval (CI) for various ETS exposure indices. Confounder-adjusted RRs for COPD were extracted, or derived using standard methods.

Meta-analyses were conducted for each exposure index, with tests for heterogeneity and publication bias. For the main index (spouse ever smoked or nearest equivalent), analyses investigated variation in RR by location, publication period, study type, sex, diagnosis, study size, confounder adjustment, never smoker definition, and exposure index definition.

**Results**: Twenty-eight relevant studies were identified; nine European or Middle Eastern, nine Asian, eight American and two from multiple countries. Five were prospective, seven case-control and 16 cross-sectional. The COPD definition involved death or hospitalisation in seven studies, GOLD stage 1+ criteria in twelve, and other definitions in nine. For the main index, random-effects meta-analysis of 33 heterogeneous (p<0.001) estimates gave a RR of 1.20 (95%CI 1.08-1.34). Higher estimates for females (1.59,1.16-2.19, n=11) than males (1.29,0.94-1.76, n=7) or sexes combined (1.10,0.99-1.22, n=15 where sex-specific not available), and lower estimates for studies of 150+ cases (1.08,0.97-1.20, n=13) partly explained the heterogeneity. Estimates were higher for Asian studies (1.34,1.08-1.67, n=10), case-control studies (1.55,1.04-2.32, n=8), and COPD mortality or hospitalisation (1.40,1.12-1.74, n=11). Some increase was seen for severer COPD (1.29,1.10-1.52, n=7). Dose-response evidence was heterogeneous. Evidence for childhood (0.88,0.72-1.07, n=2) and workplace (1.12,0.77-1.64, n=4) exposure was limited, but an increase was seen for overall adulthood exposure (1.20,1.03-1.39, n=17). We discuss study weaknesses that may bias estimation of the association of COPD with ETS.

**Conclusions**: Although the evidence strongly suggests that ETS increases COPD, study weaknesses and absence of well-designed large studies preclude reliable effect estimation. More definitive evidence is required.

## Introduction

This systematic review aims to present an up-to-date meta-analysis of available epidemiological evidence relating exposure to environmental tobacco smoke (ETS) from cigarettes to risk of chronic obstructive pulmonary disease (COPD) in lifelong non-smokers (“never smokers”). As described below, this review considers data from 28 longitudinal, case-control or cross-sectional studies ^1– 28^.

It is long established that active smoking causes COPD, the U.S. Surgeon General concluding in 1964 ^29^ that “cigarette smoking is the most important of the causes of chronic bronchitis in the United States, and increases the risk of dying from chronic bronchitis”. This opinion was echoed in their 2004 report ^30^, which felt the evidence “sufficient to infer a causal relationship between active smoking and chronic obstructive pulmonary disease morbidity and mortality”, a view confirmed by a recent systematic review ^31^.

Sidestream smoke (released between puffs from the burning cone) contains similar chemicals to mainstream smoke (drawn and inhaled by smokers), but with different relative and absolute quantities of many individual constituents ^32^. However, sidestream smoke, after mixing with aged exhaled mainstream smoke, is diluted massively by room air before non-smokers inhale it. Smoke constituent levels in tissues of non-smokers are very much lower than in smokers, studies using cotinine typically indicating a relative exposure factor between 0.06% and 0.4% ^33– 35^, with studies using particulate matter indicating a lower factor of 0.005% to 0.02% ^36– 44^. Though an effect of ETS on COPD risk is plausible, it is difficult to establish this with certainty, as a threshold is a logical possibility. The same difficulty of establishing effects of ETS exposure on other diseases caused by smoking is also present, notably for lung cancer ^31, 45^.

In 2006, a review by the U.S. Surgeon General of the association of COPD with ETS exposure ^46^ concluded that “the evidence is suggestive but not sufficient to infer a causal relationship between second-hand smoke exposure and risk for COPD”, the need for additional research also being highlighted. Although that review cited only nine of the 28 studies considered here ^1, 3, 5– 10, 13^, and although various new studies have appeared since then, no other fully comprehensive review of this subject appears to have been undertaken.

This review, which is essentially an update of the 2006 review ^46^ is an attempt to assess the epidemiological evidence currently available, restricting attention to studies of COPD in which its relationship to one or more ETS exposure indices has been studied in never smokers. This restriction to never smokers is necessary as there is a very strong association of COPD with smoking ^46^, and it is difficult to reliably detect any ETS effect where a history of smoking is present. This is because the extent of a smoker’s overall exposure to smoke constituents is determined largely by his own smoking habits and hardly at all by his much smaller ETS exposure, and also because smoking and ETS exposure are correlated (e.g. since smokers tend to marry smokers). Any errors in assessing smoking history are therefore likely to cause a residual confounding effect much larger than any plausible ETS effect ^47^.

As the 2006 US Surgeon General’s Report ^46^ notes “COPD is a non-specific term, defined differently by clinicians, pathologists, and epidemiologists, each using different criteria based on symptoms, physiologic impairment, and pathologic abnormalities”. That report goes on to state that “the hallmark of COPD is the slowing of expiratory airflow measured by spirometric testing, with a persistently low FEV _1_ [forced expiratory volume in one second] and a low ratio of FEV _1_ to FVC [forced vital capacity] despite treatment”. International guidelines ^48^ define COPD as post-bronchodilator FEV _1_/FVC <0.70, with severity classified by subdividing FEV _1_ as a percentage of predicted into four groups (≥80, <80, <50 and <30%). The term COPD was little used until the 1980s, and diagnoses commonly used earlier (e.g. chronic bronchitis and emphysema) do not correspond exactly to what is now termed COPD. The studies we selected for review used disease definitions close enough to COPD as now defined to reasonably allow overall assessment. Some studies present additional results using criteria corresponding to severer forms of the disease. While these data are presented here, they are not included in our detailed meta-analyses.

## Materials and methods

This systematic review was conducted according to PRISMA guidelines ^49^.

### Study inclusion and exclusion criteria

Attention is restricted to epidemiological longitudinal, case-control or cross-sectional studies which provide risk estimates for never (or virtually never) smokers for any of the following indices of ETS exposure: spouse, partner, cohabitant, at home, at work, in adulthood, in childhood.

The term COPD is relatively recent, so we also included studies with outcomes described otherwise. Following the strategy used in our review of smoking and COPD ^31^, outcomes “could be based on International Classification of Diseases (ICD) codes, on lung function criteria, on a combination of lung function criteria and symptoms, or on combinations of diagnosed conditions….where diagnoses were extracted from medical records or reported in questionnaires”. Acceptable combinations of diagnosed conditions had to include both chronic bronchitis and emphysema, but could also additionally include asthma, acute and unqualified bronchitis or bronchiectasis. However, studies were rejected where results were only available for emphysema, for chronic bronchitis, for respiratory symptoms such as cough or phlegm, or for lung function criteria not equating to COPD. Over-broad definitions such as respiratory disease were also not accepted. Acceptable lung function criteria included those of the Global Initiative for Chronic Obstructive Lung Disease (GOLD), the European Respiratory Society, and the British and American Thoracic Societies,

Studies which provide near equivalent definitions of “never smokers” are also accepted; thus never smokers can include occasional smokers or smokers with a minimal lifetime duration of smoking or number smoked. Risk estimates may be based on relative risks (RRs), hazard ratios (HRs), or odds ratios (ORs), and must either be provided directly or be capable of being estimated from the data provided.

### Literature searches

A PubMed search identified papers published up to June 2016 using the term “COPD AND (ENVIRONMENTAL TOBACCO SMOKE OR PASSIVE SMOKING OR SECONDHAND SMOKE EXPOSURE OR INVOLUNTARY SMOKING)”, with restriction to humans. After rejecting papers that were clearly irrelevant based on the abstract, copies of the others were obtained for inspection. Other potentially relevant papers were obtained from reference lists in the 2006 Surgeon General report ^46^, an earlier review we conducted ^47^ and relevant review papers identified in the search. The complete list of potentially relevant papers were then looked at in detail to determine those which described studies satisfying the selection criteria, the rejected papers also including those where an alternative paper provided results from the same study that were more useful (e.g. based on a longer follow-up, a larger number of cases, or using a disease definition closer to COPD as currently defined).

### Data recorded

Details were extracted from relevant publication on the following: study author; year of publication; study location; study design; sexes included; disease definition; number of cases; potential confounding variables considered; and never smoker definition. An effect estimate together with its associated 95% confidence interval (CI) was obtained, where available, for ETS exposure at home, at work, in adulthood, childhood, and from these sources combined. Choice between multiple definitions of COPD followed the rules of Forey *et al.* ^31^, except that here we also obtained additional estimates, if available, for severer COPD. We preferred effect estimates where the denominator was with no (or minimal) exposure to the ETS type considered rather than with no exposure to any ETS. Effect estimates and 95% CIs extracted were sex-specific, if possible, and for longitudinal studies were for the longest follow-up available. Estimates adjusted for covariates, where available, were generally preferred to unadjusted estimates, except that results adjusted for symptoms or precursors of COPD were not considered. Where a study provided multiple adjusted estimates, we used that adjusted for most covariates. Dose-response data were also extracted, where available.

### Derivation of effect estimates

For a study reporting effect estimates and CIs only by exposure level, that for the overall unexposed/exposed comparison was estimated using the Morris and Gardner method ^50^ for unadjusted data or the Hamling *et al.* method ^51^ for adjusted data. These methods also allowed estimation of the significance of dose-related trends, if not given in the source publication.

### Alternative types of effect estimates

As the great majority of effect estimates were ORs derived from case-control or cross-sectional studies, and as the RRs or HRs from longitudinal studies were all based on low incidences, where the OR would be virtually the same, all estimates were treated as if they were ORs. In the rest of this paper, we use OR rather than referring to specific types of effect estimate.

### Meta-analyses

A pre-planned set of fixed-effect and random-effects meta-analyses were carried out using standard methods ^52^. Heterogeneity was quantified by H, the ratio of the heterogeneity chi squared to its degrees of freedom. The I-squared statistic ^53^ is related to H by the formula I ^2^ = 100 (H-1)/H. Publication bias tests were also conducted using the Egger method ^54^.

Our main analyses included OR estimates for the exposure most closely equivalent to “spouse ever smoked” where results were provided or could be estimated. This selection was based on the source of exposure (spouse highest preference, then partner, cohabitant, home or work). Spousal smoking is traditionally used for studying possible ETS effects, it being clearly demonstrated that women married to a smoker have much higher cotinine levels than women married to a non-smoker ^55^. Apart from the meta-analyses using all available estimates, meta-analyses also investigated variation in the OR according to a list of pre-defined factors, and using the following subsets: continent (North America, Asia, Europe, multicountry); publication period (1976–1990, 1991–2005, 2006–2016); study type (longitudinal, case-control, cross-sectional); sex (males, females, combined); diagnosis (mortality or hospitalisation, GOLD stage 1+, other); method of taking asthma into account (included as part of the COPD definition, adjusted for, asthmatic participants excluded, ignored); number of cases estimate based on (<50, 50–149, 150+ cases); extent of confounder adjustment (unadjusted for age, adjusted for age and at most four other variables, adjusted for age and five or more variables); never smoker definition (never smoked any product, never smoked but product unstated, other – including never cigarette smoker, occasional smoker or very short-term smoker); and definition of exposure index (spouse specifically, other exposure at home, other).

Meta-analyses were also carried out for the main index using the estimates for severer COPD, and also for other indices of exposure with sufficient data (workplace, overall adult – including at least home and work, childhood). Here, data were too limited to study variation in the OR by the subsets described above.

Results of the overall meta-analyses are displayed as forest plots. In each plot, individual estimates are listed in increasing order of the OR. For the main index, estimates are grouped by region. Random-effects estimates are also shown. The estimates are not only shown numerically, but in graphical form on a logarithmic scale, where the OR is shown as a square, the area of which is proportional to its inverse-variance weight. Arrows warn when the CI goes outside the range of the plot.

### Study quality and risk of bias

We did not attempt to derive any overall score based on study quality and risk of bias for each individual study, as the relative importance of different sources of bias or poor study quality is difficult or impossible to assess accurately. Instead, we attempted to gain insight into this in two ways. First, as mentioned in the previous section, we carried out meta-analyses showing how the OR varied by some relevant aspects linked to study quality and bias, such as study size, study type, source of diagnosis, method of taking asthma into account, and extent of confounder adjustment. Second, we considered factors affecting quality and bias in the discussion section, including some factors that affected all or virtually all of the studies.

## Results

### Searches

**Figure 1.**
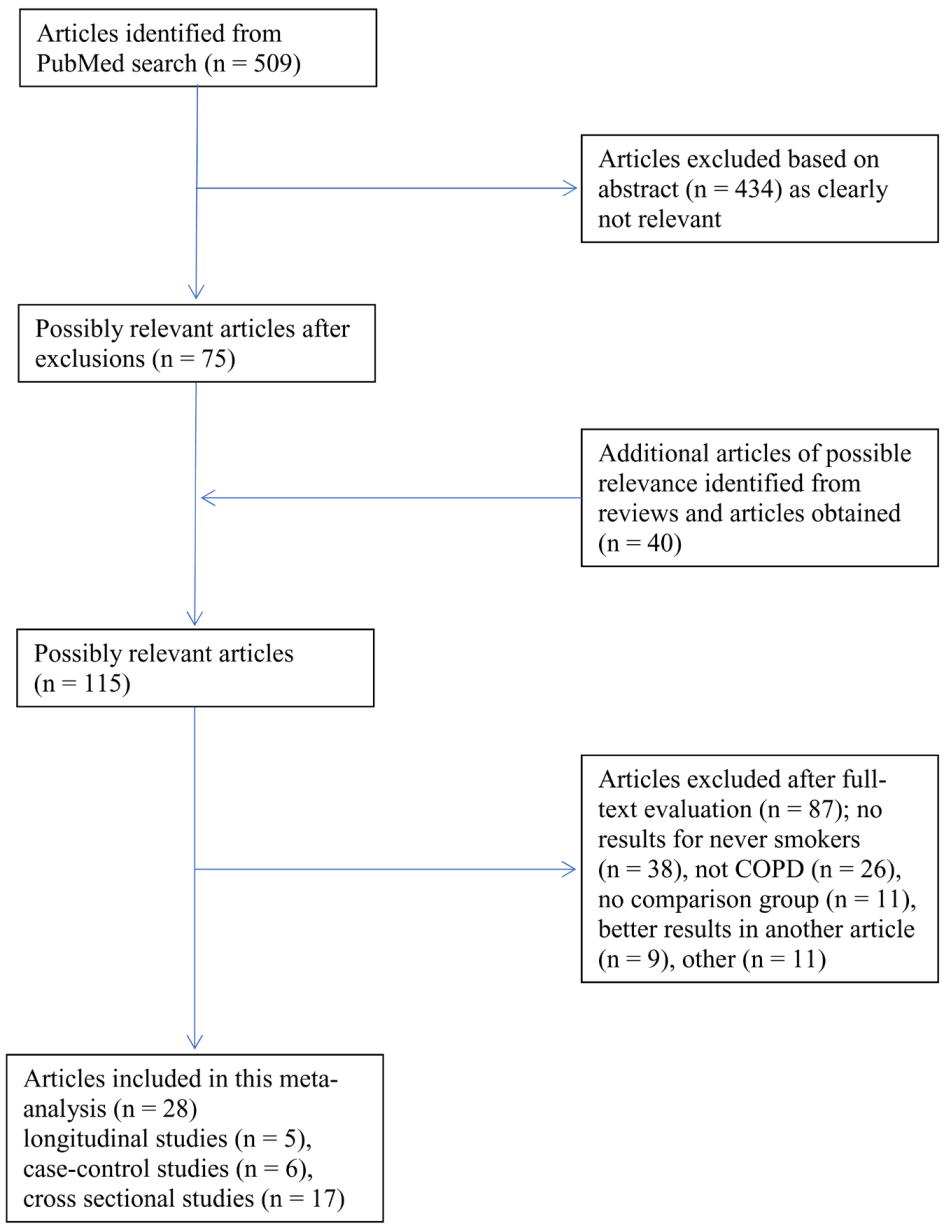
Flow-chart of study selection and exclusion criteria. The flow-chart shows the number of articles identified from the PubMed search and from reference lists of reviews and articles obtained, as well as showing those excluded, with reasons for exclusion. Note that some articles were excluded for multiple reasons.

The PubMed search produced 509 hits. As summarized in Figure 1, Seventy-five were considered of potential relevance based on the abstracts, 15 of which proved to meet the inclusion criteria on examination of the papers themselves. Further examination of reference lists in reviews ^46, 47, 56– 63^ and in papers obtained identified a further 40 papers of potential relevance, 13 of which met the inclusion criteria. Of the 87 papers examined but not accepted, the most common reasons for rejection were no results for never smokers (38 papers), not COPD as defined (26), no control group or no results for unexposed participants (11) and better results for the same cohort given in another paper (9), some studies being rejected for more than one reason. Supplementary File 1 gives details of the studies rejected and fuller reasons for rejection.

### Studies identified

Table 1 gives details of the 28 epidemiological studies that met the inclusion criteria, including author, reference(s), publication year, location, design, sexes included, disease definition, account taken of asthma, and numbers of cases in never smokers. The studies are listed in chronological order of publication and are given consecutive identifying study numbers.

**Table 1.** Studies providing evidence on COPD and ETS exposure in never smokers.

Study No.	Author ^a^	Year ^b^	Location	Type ^c^	Sexes included	Age range ^d^	Definition of COPD used ^e^	Accounting for asthma	No. of cases ^f^
1	Lebowitz ^1^	1976	USA	CS	M,F	15+	Asthma, bronchial trouble or emphysema (physician diagnosis, questionnaire report)	Included	246
2	Comstock ^2^	1981	USA	CS	M ^g^	20+	FEV _1_/FVC <0.70 (spirometry test ^h^)	Ignored	30
3	Hirayama ^3^	1984	Japan	L15	F	40+	Emphysema or chronic bronchitis (mortality)	Ignored	130
4	Krzyzanowski ^4^	1986	Poland	L13	M,F	19–70	FEV1 <65% predicted (spirometry test ^h^)	Ignored	37
5	Lee ^5^	1986	England	CC	M,F	35–74	Chronic bronchitis ^i^ (hospitalisation)	Ignored	26
6	Kalandidi ^6^	1987	Greece	CC	F	40–79	Chronic obstructive lung disease (hospitalisation)	Excluded ^j^	103
7	Sandler ^7^	1989	USA	L12	M,F	16+	Emphysema or bronchitis (mortality)	Ignored	19
8	Dayal ^8^	1994	USA	CS ^k^	M,F	Adults	Chronic bronchitis, emphysema or asthma (diagnosis, questionnaire report)	Included	219
9	Forastiere ^9^	2000	Italy	CS ^l^	F	25–74	COPD (physician diagnosis, questionnaire report)	Ignored	50
10	Enstrom ^10^	2003	USA	L39	M,F	31+	COPD (mortality)	Included	264
11	De Marco ^11^	2004	16 countries	CS	M,F	20–44	COPD (GOLD stage 1+) ^h^	Ignored	156
12	Celli ^12^	2005	USA	CS	M,F	30–80	FEV _1_/FVC <0.70 (spirometry test ^h^)	Ignored	414 ^m^
13	McGhee ^13^	2005	Hong Kong	CC	M,F	60+	COPD (including pulmonary heart disease, mortality)	Ignored	138
14	Sezer ^14^	2006	Turkey	CC	F	38+	COPD (specialist clinic diagnosis)	Ignored	74
15	Xu ^15^	2007	China	CC	M,F	35+	COPD (hospital diagnosis of emphysema or chronic bronchitis, questionnaire report)	Ignored	1097
16	Yin ^16^	2007	China	CS	M,F	51+	COPD (GOLD stage 1+ but without bronchodilator)	Excluded	429
17	Zhou ^17^	2009	China	CS	M,F	40+	COPD (GOLD stage 1+) ^n, o^	Ignored	644
18	Wu ^18^	2010	Taiwan	CC	F	40+	COPD (GOLD stage 1+)	Excluded	168
19	Jordan ^19^	2011	England	CS	M,F	40+	COPD (GOLD stage 1+ but without bronchodilator) ^p^	Excluded	779 ^m^
20	Lamprecht ^20^	2011	14 countries	CS	M,F	40+	COPD (GOLD stage 1+) ^n, q^	Adjusted	523
21	Chen ^21^	2012	China	CS	M,F	60+	COPD (physician diagnosis, questionnaire report)	Ignored	149
22	He ^22^	2012	China	L17	M,F	51–87	COPD (mortality or GOLD stage 1+) ^r^	Ignored	36
23	Waked ^23^	2012	Lebanon	CS	M,F	40+	COPD (GOLD stage 1+)	Ignored	25
24	Moreira ^24^	2013	Brazil	CS	F	40+	COPD (GOLD stage 1+)	Excluded	43
25	Eze ^25^	2014	Switzerland	CS	M,F	18–65	COPD (GOLD stage 1+) ^h^	Ignored	444
26	Hagstad ^26^	2014	Sweden	CS	M,F	20–77	COPD (GOLD stage 1+)	Ignored	41 ^m^
27	Kim ^27^	2014	Korea	CS	M,F	40+	COPD (GOLD stage 1+ but without bronchodilator)	Adjusted	323
28	Tan ^28^	2015	Canada	CS	M,F	40+	COPD (LLN, FEV1/FVC <5th centile) ^s^	Adjusted	161

^a^ First author and reference of principal publication ^b^ Year of publication ^c^ Study types are CC = case-control, CS = cross-sectional, L = longitudinal. For longitudinal studies, number of years follow-up is shown ^d^ Age at baseline for longitudinal studies ^e^ Definition of principal COPD outcome is shown. Definition of severer COPD used is shown in footnotes, along with alternative definitions for which results are available ^f^ Number of cases in lifelong non-smokers ^g^ Study also included females, but none had COPD ^h^ No mention of use of bronchodilator prior to spirometry ^i^ Named as chronic bronchitis, but defined by authors as ICD 9 ^th^ revision 491, 492, 496 so equates to COPD ^j^ Ignored for controls ^k^ Analysed as a nested CC study ^l^ Never smoking women had been identified by earlier studies in the same areas ^m^ Approximate estimate ^n^ Severer outcome definition based on GOLD Stage 2+ ^o^ Alternative results are also available for GOLD stage 0+ ^p^ Severer outcome definition based on NICE criteria (FEV _1_/FVC <0.7 and FEV _1_ <80% predicted) described as equivalent to GOLD stage 2+ (no bronchodilator, omitting participants with diagnosis of asthma). Alternative results are also available based on the LLN criteria, for “clinically significant COPD” based on the LLN, GOLD and NICE criteria, and including participants with diagnosis of asthma ^q^ Alternative results are also available using the LLN criteria ^r^ Based on death certificate, supplemented by medical records for lung function if their death was not from COPD ^s^ Severer outcome definition is based on LLN criteria plus FEV _1_<80% predicted

The included studies are mainly of representative populations, except that studies 18 and 26 have a large proportion with respiratory symptoms. Of the 28 studies, one was published in the 1970s, six in the 1980s, one in the 1990s, nine between 2000 and 2009 and 11 more recently.

Nine studies were conducted in Europe or the Middle East, subsequently referred to as “Europe” (two in England, and one each in Greece, Italy, Lebanon, Poland, Sweden, Switzerland and Turkey), while nine took place in Asia (five in China, and one each in Hong Kong, Japan, Korea and Taiwan), seven in North America (six in the USA and one in Canada) and one in South America (Brazil). Two studies presented combined results, one from 16 countries, the other from 14.

Five studies were longitudinal in design, with the length of follow-up varying from 12 to 39 years, one was a cross-sectional study analysed as a nested case-control study, 16 other studies were cross-sectional, with the remaining six of case-control design.

Most studies were of both sexes, though six studies considered only females.

Two studies considered those with a minimum age of 60, with a further 16 having a minimum age between 35 and 51. Other studies had a lower minimum age.

Definitions of outcome used varied by study. Seven studies required the case to have died or been hospitalised for COPD, while a further 12, mainly relatively recent cross-sectional studies, used COPD as defined by the GOLD stage 1+ criteria. The remaining nine studies used other definitions, as detailed in Table 1. Five studies (17, 19, 20, 26, 28) also provided results for severer COPD (generally equivalent to GOLD 2+, see footnotes to Table 1).

Twenty-one studies ignored asthma in their outcome definition and analysis, with the remaining 12 studies equally divided into those that included asthma in their outcome definition, excluded asthmatics, or adjusted for asthma status in analysis.

Most studies were small, with ten studies considering less than 100 cases and only one study (15) more than 1000 cases.

Table 2 gives the adjustment variables used and the definitions of never smokers used in the studies.

**Table 2.** Potential confounding variables adjusted for and definition of never smoker.

Study No.	Author	Variables adjusted for	Definition of never smokers ^a^
1	Lebowitz	None	Never NOS
2	Comstock	Age, education, number of bathrooms, persons/room, children in household, air conditioning, cooking fuel	Never cigarettes
3	Hirayama	Age of husband	Never cigarettes
4	Krzyzanowski	Age	Never NOS
5	Lee	Age, marital status ( Table 3) Age ( Table 5, Table 6 and Table 7)	Never NOS
6	Kalandidi	Age, occupation	Never NOS
7	Sandler	Age, housing quality, schooling, marital status	Never any product
8	Dayal	Age, sex, neighbourhood, heating, cooking	Never NOS
9	Forastiere	Age, center, age x center, education	Never cigarettes
10	Enstrom	Age ^b^	Never any product ^c^
11	De Marco	Sex, childhood respiratory infections, occupational exposure, socioeconomic status	Never smoked 20 packs of cigarettes or 360 g of tobacco in a lifetime, or at least 1 cigarette/day or 1 cigar/week for a year
12	Celli	Age, sex, race/ethnicity, BMI, education, poverty, urban residence, high risk industry, high risk occupation, biomass, allergy	Never smoked 100 cigarettes in lifetime
13	McGhee	Age, education ( Table 3) Age, sex, education ( Table 4)	Never NOS
14	Sezer	Wood ash, biomass ^d^	Never NOS
15	Xu	Education, occupation, family income, cooking fuels, heating in winter, ventilating fans, occupational physical activity	Never NOS
16	Yin	Age, sex, education, occupational dust exposure, indoor air pollution	Never NOS
17	Zhou	Age, sex, education, BMI, family history of respiratory disease, biomass, heating fuel, ventilation in kitchen, childhood chronic cough, occupational exposures	Never NOS
18	Wu	Age, height, education level, cooking status, burning incense, tea consumption	Never smoked, on average, more than 1 cigarette/day for a year
19	Jordan	Age, sex, year of study	Never smoked at least 1 cigarette/day
20	Lamprecht	None (COPD in Table 3) Age, education, occupational exposure, biomass fuel use, childhood hospitalisation, comorbidity, BMI (severer COPD in Table 3)	Never smoked more than 20 packs in lifetime or more than 1 cigarette/day for a year
21	Chen	None	Never cigarettes
22	He	Age, sex, marital status, occupation, education, alcohol, diastolic blood pressure, triglyceride and total cholesterol levels, BMI	Never smoked 100 cigarettes in lifetime
23	Waked	Age, sex, area of residence	Never NOS
24	Moreira	None	Never NOS
25	Eze	None	Never NOS
26	Hagstad	Age, sex, asthma, family history of obstructive airway disease, socioeconomic group (COPD in Table 3) None (severer COPD in Table 3 and Ever home/work and Childhood in Table 6)	Never smoked more than 1 cigarette/day for a year
27	Kim	Age, sex, previous diagnosis of asthma or tuberculosis, family income, education status	Never cigarettes
28	Tan	Age, education, childhood respiratory illness, heart disease, hypertension or diabetes, asthma, BMI, exposure to organic dust, inorganic dust, gases and vapours, biomass cooking and heating for ≥ 10 years, TB	Never smoked more than 1 cigarette/day for a year

^a^ Never any product = never smoked cigarettes, pipes or cigars; Never NOS = never smoked, product unspecified; ^b^ Results adjusted for more variables not used as adjustment included health status ^c^ Questions on pipe and cigar smoking were asked at baseline, but not at the follow-up interviews ^d^ The cases and controls were matched on age

Five studies (1, 20, 21, 24, 25) made no adjustment for any potential confounding variables, while some others made little or no adjustment for such variables as occupation, education, diet and family history of disease, which may differ between smoking and non-smoking households ^64^. Failure to adjust for household size, where the index of exposure is based on presence of a smoker in the household, was also common. Where adjustment was carried out, all but four studies considered age, although study 3 adjusted for the husband’s age rather than the subject’s.

Fifteen studies were of never smokers, though only three of these made it clear they were never smokers of cigarettes, pipes or cigars. Five studies were of never cigarette smokers (i.e. they may have included some pipe or cigar only smokers), the remaining eight allowing a minimal smoking history, such as smoking less than 1 cigarette a day or less than 100 cigarettes in life.

### Main exposure index

The main meta-analyses use an exposure index that relates as closely as possible to ever smoking by the spouse. Table 3 shows the definitions of ETS exposure used for the main index. This was based on smoking by the spouse for five studies, and on smoking by cohabitants for a further 13 (although study 13 only included participants who had lived with a smoker 10 years previously, and study 20 only considered ETS exposure in the home in the two weeks prior to the study). For the remaining studies, the index was based on exposure in the home and at work (studies 4, 12, 17, 18 and 27) or on a combination of exposure from any source (studies 11, 15, 19, 21 and 25).

**Table 3.** COPD among never smokers and smoking by the spouse or household member ^a^.

Study No.	Author	Type ^b^	Sex	Definition of exposure ^c^	Number of cases		Odds ratio (95% CI) ^d^
					Unexposed	Exposed	
COPD:							
1	Lebowitz	CS	M+F	Lives with ever smoker ^e^	129	117	1.09 (0.83-1.44) ^f^
2	Comstock	CS	M	Lives with a smoker	23	7	1.19 (0.50-2.86) ^f^
3	Hirayama	L15	F	Husband ever smoked	28	102	1.38 (0.86-2.21) ^f^
4	Krzyzanowski	L13	F	Exposure at home or workplace	26	6	0.36 (0.15-0.86) ^f^
			M		3	2	1.39 (0.26-7.40) ^f^
5	Lee	CC	F	Spouse smoked in marriage	4	13	1.22 (0.38-3.94) ^f^
			M		8	1	0.34 (0.06-2.03) ^f^
6	Kalandidi	CC	F	Husband ever smoked ^e^	13	90	1.38 (0.69-2.76) ^f^
7	Sandler	L12	F	Lived with a smoker	2	11	5.65 (1.19-26.8)
			M		4	2	0.93 (0.16-5.32)
8	Dayal	CS	M+F	Lives with a smoker	74 ^g^	145 ^g^	1.40 (0.98-1.99) ^f^
9	Forastiere	CS	F	Ever married to a cigarette smoker	11	39	1.75 (0.88-3.47)
10	Enstrom	L39	F	Spouse ever smoked ^e^	45	128	1.13 (0.80-1.58)
			M		69	22	1.27 (0.78-2.08)
11	De Marco	CS	M+F	4+ hours per day exposure on most days/nights in previous 12 months	129	27	1.14 (0.74-1.77) ^f^
12	Celli	CS	M+F	Lives with a smoker who smokes in the home, or exposed at work at least 1 hour per day	327 ^g^	86 ^g^	0.88 (0.57-1.36)
13	McGhee	CC	F	Lived with a smoker 10 yrs ago	15	27	2.90 (1.34-6.29)
			M		69	27	1.67 (0.95-2.94)
14	Sezer	CC	F	Lived with a smoker for at least 10 yrs	13	61	2.57 (1.04-6.36) ^f^
15	Xu	CC	M+F	Spent 15+ minutes, 3+ times per week in room with smoker at any time in life	Total 1097		0.95 (0.79-1.16)
16	Yin	CS	M+F	Lived with a smoker	195	234	0.95 (0.77-1.18) ^f^
17	Zhou	CS	M+F	Exposure at home or workplace	119 ^g^	525 ^g^	1.34 (1.08-1.65) ^f^
18	Wu	CC	F	Exposure at home (including childhood) or workplace	41	127	2.20 (1.39-3.49) ^f^
19	Jordan	CS	M+F	1+ hours of exposure per week	Total 779 ^g^		1.11 (0.95-1.30) ^f^
20	Lamprecht	CS	M+F	Exposure at home in previous 2 weeks	423	100	0.89 (0.70-1.20) ^f^
21	Chen	CS	M+F	Exposure at home, workplace or other places	85	64	1.82 (1.30-2.54) ^f^
22	He	L17	M+F	Exposure at home for 15+ minutes per day, 1+ days per week for 2+ years ^h^	10	4	1.67 (0.49-5.78)
23	Waked	CS	M+F	Lives with a smoker	Total 25		1.23 (0.55-2.74) ^f^
24	Moreira	CS	F	Lives with a smoker	Total 43		No significant difference in number of years exposure, or proportion exposed to straw cigarettes ^i^
25	Eze	CS	M+F	Exposure at home, workplace or other places in previous year	293	151	1.00 (0.81-1.24) ^f^
26	Hagstad	CS	M+F	Ever lived with a smoker ^j^	25	64	1.38 (0.84-2.27)
27	Kim	CS	M+F	Exposure at home or workplace	248	75	0.85 (0.60-1.21) ^f^
28	Tan	CS	F	Lived with a smoker in previous 2 weeks	M&F 94	M&F 12	2.20 (1.03-4.71)
			M				1.01 (0.27-3.76)
Severer COPD ^k^:							
17	Zhou	CS	M+F	Exposure at home or workplace ^j^	89	379	1.27 (1.00-1.63) ^f^
19	Jordan	CS	M+F	1+ hours of exposure per week	Total 334 ^g^		1.13 (0.84-1.51) ^f^
20	Lamprecht	CS	F	Exposure at home in previous 2 weeks	Total 159		1.53 (0.98-2.41)
			M		Total 67		0.97 (0.40-2.40)
26	Hagstad	CS	M+F	Ever lived with a smoker ^j^	11	35	2.46 (1.24-4.88) ^f^
28	Tan	CS	F	Lives with a smoker	M&F 94	M&F 4	1.65 (0.46-5.88)
			M				0.69 (0.08-6.31)

^a^ Or nearest equivalent to spouse or household member (see text and Table) ^b^ Study types are CC = case-control, CS = cross-sectional, L = longitudinal. For longitudinal studies, number of years follow-up is shown ^c^ Comparison is with those not exposed as defined, except where indicated otherwise ^d^ RRs from longitudinal studies are taken as being equivalent to ORs ^e^ Separate results also available for current smoker and exsmoker ^f^ OR and/or CI estimated from data provided ^g^ Approximate estimates ^h^ Compares exposed at home only to unexposed. Excludes those exposed at work ^i^ A straw cigarette is a handful of tobacco, wrapped in a corn husk; study not included in meta-analysis ^j^ Compared to subjects not exposed to any source of ETS; results also available for current or former exposure ^k^ Results not included in the meta-analysis in Figure 2

Although most studies presented results comparing participants who were exposed or unexposed to ETS, some required a minimum level before a subject could be classified as exposed. In study 19, exposure had to be for at least one hour per week, while study 12 specified living with a smoker who smoked in the home or exposure at work for at least one hour per day. In studies 20 and 28, exposure had to have been in the previous two weeks, while participants in study 25 had to have had regular exposure in the previous year. In study 22 exposure had to be for 15+ minutes per day at least once per week for two or more years, while in study 15 the minimum requirement was 15 minutes or more, three or more times per week. In study 11, participants were only considered to have been exposed if they reported four or more hours of exposure on most days or nights in the previous year. Finally, study 14 required 10 years of exposure.

**Figure 2.**
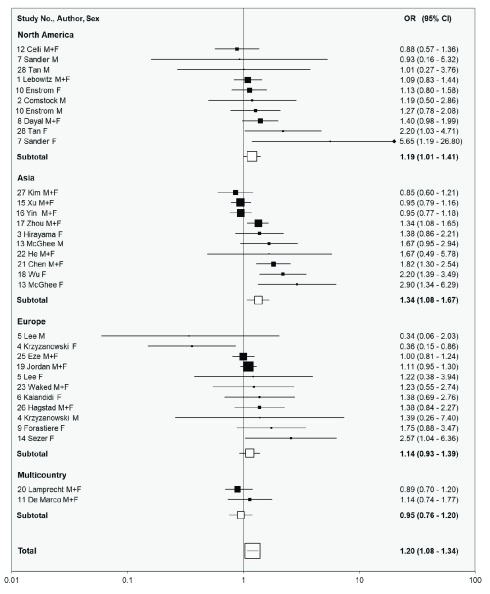
Forest plot for the main index, by region. Individual study estimates of the OR and its 95%CI are shown separately by region, sorted in increasing order of OR. These are shown as numbers, and also graphically on a logarithmic scale. Random-effects estimates of ORs and 95%CIs are also shown for each region combined and overall. Studies are identified by the study number shown in Table 1. In the graphical representation, ORs are indicated by a square, with the area of the square proportional to the weight.

Table 3, supported by Figure 2, also presents the ORs for the main exposure index, while Table 4 presents the results of meta-analyses, and Table 5 the dose-response data.

**Table 4.** Meta-analyses of COPD ^a^ risk among never smokers and smoking by spouse or household member ^b^.

		Fixed-effect	Random- effects	Publication bias	Heterogeneity ^c^	
Subgroup	N ^d^	Odds ratio (95% CI)	Odds ratio (95% CI)	p ^e^	Chisquared per DF ^f^	p ^g^
All COPD	33	1.14 (1.07-1.21)	1.20 (1.08-1.34)	<0.1	1.99	<0.001
*By continent*						
North America	10	1.19 (1.02-1.38)	1.19 (1.01-1.41)	NS	1.08	NS
Asia	10	1.18 (1.07-1.30)	1.34 (1.08-1.67)	<0.1	3.83	<0.001
Europe ^h^	11	1.10 (0.98-1.24)	1.14 (0.93-1.39)	NS	1.53	NS
Multicountry	2	0.95 (0.76-1.20)	0.95 (0.76-1.20)	-	0.90	NS
			*Between* *continents*		*1.13*	*NS*
*By publication period*						
1976–1990	10	1.12 (0.91-1.37)	1.11 (0.81-1.52)	NS	1.52	NS
1991–2005	8	1.28 (1.09-1.50)	1.30 (1.07-1.59)	<0.1	1.40	NS
2006–2015	15	1.11 (1.03-1.20)	1.19 (1.03-1.37)	<0.1	2.70	<0.001
			*Between periods*		*1.18*	*NS*
*By study type*						
Longitudinal	8	1.18 (0.94-1.47)	1.18 (0.83-1.67)	NS	1.71	NS
Case-control	8	1.20 (1.02-1.40)	1.55 (1.04-2.32)	NS	3.36	<0.01
Cross-sectional	17	1.12 (1.04-1.21)	1.14 (1.02-1.27)	NS	1.72	<0.05
			*Between types*		*0.34*	*NS*
*By sex*						
Males	7	1.29 (0.94-1.76)	1.29 (0.94-1.76)	NS	0.55	NS
Females	11	1.50 (1.25-1.81)	1.59 (1.16-2.19)	NS	2.40	<0.01
Both	15	1.08 (1.01-1.16)	1.10 (0.99-1.22)	NS	1.81	<0.05
			*Between sexes*		*5.57*	*<0.01*
*By diagnosis*						
Mortality ^i^ or hospitalisation	11	1.37 (1.13-1.66)	1.40 (1.12-1.74)	NS	1.13	NS
GOLD Stage 1+	10	1.09 (1.01-1.19)	1.11 (0.97-1.27)	NS	2.22	<0.05
Other	12	1.15 (1.02-1.29)	1.23 (0.97-1.56)	NS	2.54	<0.01
			*Between* *diagnoses*		*2.24*	*NS*
*By method for taking* *asthma into account*						
Included	4	1.19 (1.00-1.41)	1.19 (1.00-1.41)	NS	*0.45*	*NS*
Adjusted for	4	0.94 (0.77-1.15)	1.00 (0.72-1.39)	NS	*1.76*	*NS*
Excluded	4	1.11 (0.99-1.26)	1.24 (0.93-1.65)	NS	*3.63*	*<0.05*
Ignored	21	1.18 (1.08-1.30)	1.27 (1.07-1.50)	NS	*2.08*	*<0.01*
			*Between* *methods*		*1.49*	*NS*
*By number of cases* *estimate is based on*						
<50	11	1.29 (0.97-1.71)	1.26 (0.83-1.92)	NS	1.85	<0.05
50–149	9	1.62 (1.35-1.96)	1.62 (1.35-1.96)	NS	0.53	NS
150+	13	1.07 (1.00-1.15)	1.08 (0.97-1.20)	NS	1.97	<0.05
			*Between* *numbers*		*8.65*	*<0.001*
*By extent of confounder* *adjustment*						
Unadjusted for age	6	1.11 (0.98-1.25)	1.15 (0.94-1.42)	NS	2.55	<0.05
Adj. for age+ <5 variables	18	1.16 (1.05-1.28)	1.26 (1.06-1.49)	NS	1.75	<0.05
Adj. for age+ 5+ variables	9	1.13 (1.00-1.27)	1.20 (0.95-1.53)	NS	2.61	<0.01
			*Between groups*		*0.16*	*NS*
*By definition of never* *smoker*						
Never any product	4	1.22 (0.93-1.61)	1.28 (0.88-1.87)	NS	1.34	NS
Never, product unstated	15	1.10 (1.01-1.20)	1.16 (0.99-1.36)	NS	2.15	<0.01
Other	14	1.17 (1.06-1.30)	1.25 (1.05-1.50)	NS	2.18	<0.01
			*Between* *definitions*		*0.63*	*NS*
*By definition of exposure*						
Spouse specifically	7	1.26 (1.02-1.55)	1.26 (1.02-1.55)	NS	0.60	NS
Other lives with smoker	15	1.14 (1.02-1.29)	1.31 (1.08-1.59)	<0.01	1.85	<0.05
Other	11	1.11 (1.03-1.21)	1.13 (0.95-1.34)	NS	3.31	<0.001
			*Between* *definitions*		*0.55*	*NS*
All severer COPD	7	1.29 (1.10-1.52)	1.29 (1.10-1.52)	NS	0.94	NS

^a^ Definition of COPD as shown in the body of Table 1, severer COPD in footnotes to Table 1. Data as shown in Table 3 ^b^ Or nearest equivalent to spouse or household member (see text and Table 3) ^c^ Heterogeneity relates to variation between studies within subgroup, except for results given in italics which relate to heterogeneity between subgroups ^d^ N number of estimates in meta-analysis ^e^ Egger test p expressed as <0.001, <0.01, <0.05, <0.1 or NS (p≥0.1) ^f^ DF degrees of freedom ^g^ p expressed as <0.001, <0.01, <0.05, <0.1 or NS (p≥0.1) ^h^ Includes one study from Turkey and one from Lebanon ^i^ Including study 22

**Table 5.** Dose-response evidence for COPD among never smokers for smoking by spouse or household member.

Study No.	Author	Type ^a^	Sex	Exposure Source	Level	No. of cases	Odds ratio (95% CI) ^b^	Trend p ^c^
COPD:								
3	Hirayama	L15	F	Husband	Never smoked Ex smoker or 1–19/day 20+/day	28 65 37	1.00 1.29 (0.79-2.12) ^d^ 1.60 (0.92-2.78) ^d^	NS
6	Kalandidi	CC	F	Husband	Never smoked Daily consumption ≤1 pack/day ≥1 pack/day Lifelong consumption ≤300,000 cigs 300,000+ cigs	13 35 37 52 38	1.00 2.5 (1.3-5.0) 1.5 (0.8-2.7) 1.30 (0.64-2.64) ^d^ 1.70 (0.72-4.03) ^d^	NS NS
8	Dayal	CS	M+F	Cohabitants	No smoker ≤1 pack/day ^f^ >1 pack/day ^f^	74 ^e^ 76 ^e^ 69 ^e^	1.00 1.16 (0.78-1.72) 1.86 (1.21-2.86)	++
10	Enstrom	L39	F M	Husband Wife	Per level ^g^ Per level ^g^	173 91	0.99 (0.92-1.06) 1.06 (0.91-1.25)	NS NS
13	McGhee	CC	M+F	Cohabitants	No smoker 1 smoker 2+ smokers	84 54 ^h^	1.00 1.85 (1.14-3.00) 2.51 (1.22-5.18)	++
14	Sezer	CC	F	Cohabitants	<10 years 10–19 years 20–29 years 30+ years	13 12 20 29	1.00 1.19 (0.58-5.68) 2.46 (0.83-7.33) 4.96 (1.65-14.86)	++
16	Yin	CS	M+F	Cohabitants	No smoker 1 smoker 2+ smokers <2 years of 40 hours/wk 2–5 years of 40 hours/wk 5+ years of 40 hours/wk	195 201 33 273 73 83	1.00 0.96 (0.77-1.20) 0.92 (0.62-1.36) 1.00 1.11 (0.84-1.47) 1.60 (1.23-2.10)	NS ++
18	Wu	CC	F	Lifetime cohabitants and co- workers	No exposure <32 years 32+ years	41 58 69	1.00 1.86 (1.10-3.17) ^d^ 2.53 (1.51-4.26) ^d^	+ ^i^
19	Jordan	CS	M+F	Any exposure	Total No exposure 1–19 hours/wk 20+ hours/wk	779 ^e^	1.00 1.11 (0.94-1.31) 1.10 (0.81-1.49)	NS
22	He	L17	M+F	Cohabitants and co- workers	Score 0 ^f^ Score 1-2 Score 3-4 Score 5-6	10 8 13 5	1.00 1.52 (0.57-4.04) 2.32 (0.98-5.50) 5.01 (1.65-15.24)	++
27	Kim	CS	M+F	Home or workplace	Total No exposure ≤6 hours/day >6 hours/day	323	1.00 0.83 (0.58-1.19) 1.75 (0.47-6.59)	NS
Severer COPD:								
19	Jordan	CS	M+F	Any exposure	Total No exposure 1-19 hours/wk 20+ hours/wk	334 ^e^	1.00 1.10 (0.81-1.49) 1.33 (0.74-2.38)	NS

^a^ Study types are CC = case-control, CS = cross-sectional, L = longitudinal. For longitudinal studies, number of years follow-up is shown ^b^ RRs from longitudinal studies are taken as being equivalent to ORs ^c^ NS p≥0.05, + p<0.05, ++ p<0.01 ^d^ OR and/or CI estimated from data provided ^e^ Approximate estimates ^f^ Sum of smoking levels for all cohabitants ^g^ For husband smoking, there were 8 levels: never, former, current pipe/cigar, and current cigs/day 1-9, 10-19, 20, 21-39 and 40+. For wife smoking there were 7 levels, as for husband except with no level for pipe/cigar ^h^ Number of cases is for the exposed groups combined ^i^ Trend estimated from data provided ^j^ Sum of scores for exposure at home (0 = no exposure, 1 = <4 pack years, 2 = 4 to <8 pack years, 3 = ≥8 pack years) and at work (0 = no exposure, 1 = <5, 2 = 5 to <15, 3 = ≥15, calculated from (pack years x smokers x hours/day)/100

From Table 3 it can be seen that, of the 33 individual OR estimates given for COPD, 24 are above 1.00, seven of these increases being significant at p<0.05. Eight studies reported an OR below 1.00, but only in study 4 for females was the reduction statistically significant. Study 25 reported an OR of 1.00, while study 24, excluded from the meta-analyses, did not present an OR but reported no significant relationship with duration or type of exposure. In addition, five studies presented a total of seven OR estimates for severer COPD, with five estimates above 1.00 (one significantly so and one marginally significant) and two non-significantly below 1.00.

Table 4 demonstrates that the overall evidence for the main exposure index shows some increased risk of COPD, with the random-effects OR, based on 33 independent estimates, being 1.20 (95% CI 1.08-1.34) with no clear evidence of publication bias (0.05<p<0.1), but clear heterogeneity (p<0.001). The largest contributors to this were the high ORs for studies 18 and 21 and in females for study 13 and the low OR in females for study 4.

Although there was no significant heterogeneity by continent, a significant increase was seen for North America (1.19, 1.01-1.41, n = 10) and Asia (1.34, 1.08-1.67, n = 10), but not for other locations. There was no significant heterogeneity by period of publication or study type. There was evidence of heterogeneity by sex (p<0.01), with a significant increase only for females (1.59, 1.16-2.19, n = 11). There was no heterogeneity by aspects of diagnosis, although the estimates were highest for definitions based on mortality or hospitalisation (1.40, 1.12-1.74, n = 11). However, there was significant heterogeneity (p<0.001) by numbers of cases, with larger ORs from studies of less than 50 cases (1.26, 0.83-1.92, n=11) and from studies of 50-149 cases (1.62, 1.35-1.96, n=9) than for studies of 150 or more cases (1.08, 0.97-1.20, n=13). There was no significant evidence of heterogeneity by extent of confounder adjustment, or by how never smokers or the exposure index were defined. For all these subgroup analyses, there was little evidence of publication bias, but evidence of heterogeneity in some subgroups.

The combined OR for severer COPD was significant (1.29, 1.10-1.52, n=7).

There was also evidence of a dose-response relationship, as shown in Table 5, with six of 11 studies investigating this reporting a statistically significant positive trend. Study 16 reported no trend in relation to the number of smokers in the household, but did report positive dose-response relationships for years of ETS exposure at home and at work. Study 19, which found no relationship with the main COPD outcome, also presented dose-response relationships for severer COPD, again finding no significant increase in risk with increasing exposure.

### Other exposure indices

Five studies also presented additional results for other indices of ETS exposure, as shown in Table 6. Four studies (16, 22, 23, 26) looked at exposure at work, all but study 23 also presenting results for combined exposure at home and at work. Study 5 produced a combined index of adulthood exposure at home or work, or during travel or leisure. Three studies (16, 23, 26) considered childhood ETS exposure, study 23 studying exposure from both the mother and the father, and also looking at parental smoking during pregnancy.

**Table 6.** Odds ratio for COPD among never smokers for other indices of ETS exposure.

Study No.	Author	Type ^a^	Sex	Number of cases		Index of exposure ^b^	Odds ratio (95% CI) ^c^	Meta- analysis ^d^
				Unexposed	Exposed			
COPD:								
5	Lee	CC	F	7	5	Combined index of adulthood exposure at home, work, during travel and leisure ^e^	1.04 (0.34-3.20) ^f^	A
			M	1	3		1.18 (0.19-7.42) ^f^	A
16	Yin	CS	M+F	225	204	Childhood – home	0.87 (0.71-1.06) ^f^	C
				240	187	Adulthood – work	0.96 (0.78-1.19) ^f^	W
				191	238	Adulthood - home and/or work ^g^	1.24 (1.01-1.51) ^f^	A
22	He	L17	M+F	10	26	Workplace ^h^	2.52 (1.00-6.38)	W
			M	8	15	Adulthood – home and/or work	2.15 (0.86-5.39)	A
			F	2	11		3.31 (0.69-15.82)	A
23	Waked	CS	M+F	Total 25		During pregnancy: mother	1.59 (0.51-4.92) ^f^	
						During pregnancy: father	1.69 (0.73-3.90) ^f^	
						Childhood: mother	1.17 (0.39-3.52) ^f^	C
						Childhood: father	1.36 (0.61-3.07) ^f^	
						Workplace	0.75 (0.18-3.14) ^f^	W
26	Hagstad	CS	M+F	25	78	Previous workplace ^i^	1.42 (0.86-2.33)	
				25	14	Current workplace ^i^	1.17 (0.58-2.36)	W
				25	10	Previous and current workplace ^i^	1.74 (0.77-3.91)	
				25	5	Ever at home and both previous and current workplace ^i^	3.80 (1.29-11.20)	
				25	3	Currently at home and both previous and current workplace ^i^	5.73 (1.46-22.50)	
				25	115	Ever at home and/or work ^i^	1.87 (1.20-2.91) ^f^	A
Severer COPD:								
26	Hagstad	CS	M+F	Total 27		Childhood ^j^	0.62 (0.28-1.35) ^f^	

^a^ Study types are CC = case control, CS = cross-sectional, L = longitudinal. For longitudinal studies, number of years follow-up is shown ^b^ Comparison is with no exposure of the type specified, except where indicated otherwise ^c^ RRs from longitudinal studies are taken as being equivalent to ORs ^d^ A = Any adult, C = Childhood and W = Workplace indicate estimates included in Table 7 meta-analysis ^e^ Comparison is with those with no exposure of any of the four types, or at most little exposure from one of them ^f^ OR and/or CI estimated from data provided ^g^ Comparison is with those with <2 years of 40 hours per week exposure ^h^ Compares exposed at work only to unexposed. Excludes those exposed at home ^i^ Comparison group is subjects not exposed to ETS from any source ^j^ From 65

**Figure 3.**
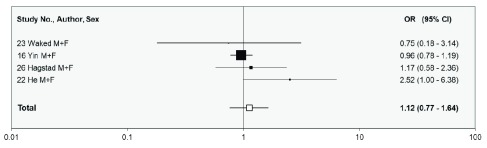
Forest plot for workplace exposure. Individual study estimates of the OR and its 95%CI are shown sorted in increasing order of OR. These are shown as numbers, and also graphically on a logarithmic scale. Random-effects estimates of ORs and 95%CIs are also shown. Studies are identified by the study number shown in Table 1. In the graphical representation, ORs are indicated by a square, with the area of the square proportional to the weight.

**Figure 4.**
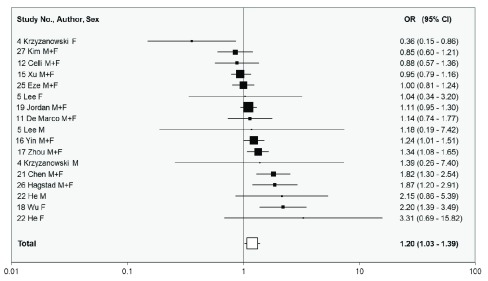
Forest plot for overall adult exposure. Individual study estimates of the OR and its 95%CI are shown sorted in increasing order of OR. These are shown as numbers, and also graphically on a logarithmic scale. Random-effects estimates of ORs and 95%CIs are also shown. Studies are identified by the study number shown in Table 1. In the graphical representation, ORs are indicated by a square, with the area of the square proportional to the weight.

The ORs for these other exposure indices are supported by Figure 3 (workplace) and Figure 4 (overall adult), while Table 7 presents the results of meta-analyses. Note that Figure 4, and the meta-analyses for overall adult exposure, consider not only the ORs indicated in Table 6, but also include estimates from Table 3 for those ten studies (4, 11, 12, 15, 17, 18, 19, 21, 25, 27) for which the exposure was at least from home and work.

**Table 7.** Meta-analyses of COPD ^a^ risk among never smokers for other indices of ETS exposure.

		Fixed-effect	Random-effects	Publication bias	Heterogeneity	
Index of exposure	N ^b^	Odds ratio (95% CI)	Odds ratio (95% CI)	p ^c^	Chi squared per DF ^d^	p ^e^
Workplace	4	1.01 (0.83-1.23)	1.12 (0.77-1.64)	NS	1.43	NS
Overall adult ^f^	17	1.16 (1.07-1.25)	1.20 (1.03-1.39)	NS	2.65	<0.001
Child ^g^	2	0.88 (0.72-1.07)	0.88 (0.72-1.07)	-	0.27	NS

^a^ Definition of COPD as shown in the body of Table 1. Data as shown in Table 6 excluding severer COPD ^b^ Number of estimates in meta-analysis ^c^ Egger test p expressed as <0.001, <0.01, <0.05, <0.1 or NS (p≥0.1) ^d^ Degrees of freedom ^e^ p expressed as <0.001, <0.01, <0.05, <0.1 or NS (p≥0.1) ^f^ Index includes “home or workplace” or combined index of any adulthood exposure. Note that this meta-analysis not only includes those estimates marked with an A in Table 6, but also includes estimates from Table 3 for studies 4, 11, 12, 15, 17, 18, 19, 21, 25 and 27 ^g^ Preferring exposure from the mother in study 23. Estimates would be 0.89 (0.74-1.08) fixed and 0.91 (0.70-1.18) random, preferring exposure from the father

Of the four ORs included in the meta-analysis of COPD for exposure at work, two were above 1.00, one of borderline statistical significance, and two were below 1.00, the combined estimate being 1.12 (0.77-1.64). Note that in study 26 there was a choice of workplace OR estimates, with the meta-analysis including that for current exposure. Using estimates for previous or ever exposure would not have affected the conclusion that there was no clear relationship of COPD to workplace ETS exposure.

Of the 17 ORs included in the meta-analysis for overall adult exposure, 12 were above 1.00, five significantly so, with one equal to 1.00, and four less than 1.00. The combined estimate of 1.20 (1.03-1.39) was also significantly increased.

There was no clear association of COPD with childhood ETS exposure, with none of the ORs shown in Table 6 being significant. Only two estimates could be included in the meta-analysis, giving an overall estimate of 0.88 (0.72-1.07).

There was no significant evidence of publication bias for workplace or adult exposure, the data being too limited to assess this for childhood exposure. However, there was evidence of heterogeneity (p<0.01) for overall adult ETS exposure.

The limited further dose-response data shown in Table 8 added little to the data already shown in Table 5.

**Table 8.** Dose-response evidence for COPD among never smokers for other indices of exposure.

Study No.	Author	Type ^a^	Sex	Exposure		No. of cases	Odds ratio (95% CI)	Trend p ^b^
				Source	Level			
5	Lee	CC	F	Combined index of adulthood exposure at home, work, during travel and leisure	Score 0-1 ^c^	7	1.00	
					Score 2-4	4	1.05 (0.29-3.75)	
					Score 5-12	1	1.03 (0.12-8.85)	NS ^d^
			M		Score 0-1	1	1.00	
					Score 2-4	2	0.83 (0.07-9.56)	
					Score 5-12	1	1.90 (0.11-32.61)	NS ^d^
16	Yin	CS	M+F	Childhood	No smoker	225	1.00	
					1 smoker	157	0.89 (0.72-1.10)	
					2+ smokers	47	0.81 (0.58-1.12)	NS
				Co-workers	No smoker	240	1.00	
					1 smoker	15	0.88 (0.51-1.52)	
					2+ smokers	172	0.97 (0.78-1.20)	NS
					<2 years of 40 hours/wk	286	1.00	
					2–5 years of 40 hours/wk	65	1.35 (1.01-1.80)	
					5+ years of 40 hours/wk	78	1.50 (1.14-1.97)	++
				Cohabitants and co-workers	<2 years of 40 hours/wk	191	1.00	
					2–5 years of 40 hours/wk	82	0.95 (0.72-1.24)	
					5+ years of 40 hours/wk	156	1.48 (1.18-1.85)	++

^a^ Study types are CC = case-control, CS = cross-sectional ^b^ NS = p≥0.05, + = p<0.05, ++ = p<0.01 ^c^ Based on sum of 0 = not at all, 1 = little, 2 = average, 3 = a lot for each source of exposure ^d^ Trend estimated from data provided

## Discussion

We rejected papers for various appropriate reasons. These included the following: failing to give results for an endpoint equivalent to COPD; giving results only for COPD exacerbation or prognosis; not presenting results for never smokers; describing studies without a control group; not presenting results for those unexposed to ETS; and presenting less useful results than reported in another publication.

Twenty-eight epidemiological studies did qualify for inclusion, and from 33 estimates of the risk of COPD associated with ever having a spouse who smoked, or the nearest equivalent ETS exposure index available, random-effects meta-analysis gave a significantly increased OR estimate of 1.20 (1.08-1.34). There was also some evidence of dose-response. While the clear relationship of smoking with COPD ^31^ makes it plausible that some effect will also be evident for ETS, one must emphasize that exposure is much less than from active smoking, as noted in the Introduction. Also, various limitations of the evidence, discussed below, make it difficult to estimate reliably the true extent of any causal relationship. However, one should also take into account the evidence of a relationship between ETS and wheezing ^46, 66^, a symptom of COPD.

### Few cases

Though four studies involved more than 500 cases, with the maximum 1097 in study 15, as many as ten of the 28 studies involved less than 100 cases, the quite small number of cases making it difficult to detect potential effects reliably.

### Publication bias

The observation that ORs are only modestly raised for studies with larger numbers of cases but are greater for smaller studies suggests the possibility of publication bias, with authors being more likely to report stronger relationships. However formal tests for publication bias ^54^ showed no clear evidence of its existence. One must note, though, that various large longitudinal studies, e.g. 67– 70, reported results relating ETS to smoking-related diseases such as lung cancer or heart disease, but did not do so for COPD. If any relationship had been seen, these studies might well have been reported.

### Misclassification of smoking status

No study validated the lifelong non-smoking status of their participants, although study 18 did verify current active and passive exposure in a random sample of participants by measuring urinary cotinine levels. As some current and past smokers deny smoking when interviewed ^71^, and as the smoking habits of spouses or household members are clearly correlated ^47^, misclassification of even a few ever smokers as never smokers can cause relevant bias ^72^, especially when, as is the case with COPD, the association with smoking is strong ^31^.

### Weaknesses in longitudinal studies

All the longitudinal studies considered involved follow-up for at least 12 years. Of the five studies, three (studies 3, 7 and 10) assumed spousal smoking was unchanged during follow-up, only studies 4 and 22 collecting information on smoking status at multiple time points.

All these studies only considered COPD deaths which occurred in the original study area.

### Inappropriate controls in case-control studies

Although three case-control studies used population controls, the remaining three used control groups unlikely to be representative of the population from which the cases derived. Studies 6 and 14 used visitors to the hospital attended by the cases, and study 13 used as a control a person identified by the informant of a death as a “living person about the same age who was well known to the informant”, the informant then being asked about the lifestyle 10 years earlier of both decedent and control.

### Weakness of cross-sectional studies

Over half of the studies were of cross-sectional design, a design limited by difficulties in determining whether ETS exposure or disease onset occurred first.

### Poor control for potential confounding variables

As noted above, some studies made little or no adjustment for variables likely to differ between smoking and non-smoking households. Though ORs for the main exposure index did not vary significantly by extent of adjustment, it should be noted that adjustment for dietary variables and education explains a substantial part of the association of lung cancer with spousal smoking ^45^. The same may be the case for COPD.

### Variation and appropriateness of diagnostic criteria

Definitions of COPD used were all consistent with the inclusion criteria. However, they still varied somewhat between study, further adding uncertainty to the meta-analysis results. Even given the inclusion criteria, there are doubts about the appropriateness of the diagnostic criteria used in some studies. In study 8, for example, the definition included asthma as well as chronic bronchitis and emphysema, the diagnosis being reported by the head of the household, and not necessarily made by a doctor.

### Misclassification of ETS exposure

While random errors in determining ETS exposure led to underestimation of the relationship of COPD with ETS, errors may not be random. Twenty-three of the 28 studies considered were of case-control or cross-sectional design, where recall bias may exist if those with COPD tend to overestimate their ETS exposure compared to those without COPD. Exposure was generally not validated by biochemical markers or air measurements taken at home.

### Limited evidence for some sources of ETS

Only 15 studies (4, 5, 11, 12, 15–19, 21–23, 25–27) provided data on ETS exposure from sources other than the home. Five (4, 12, 17, 18, 27) presented results only for a combined household and workplace exposure index, with a further five (11, 15, 19, 21, 25) only presenting results for total exposure irrespective of location, results used in our analyses as the nearest equivalent which was available to smoking by the spouse or household member. While there are far less available data on risk of COPD from ETS exposure specifically in the workplace or in childhood than on smoking in the home, the available data show no clear relationship of risk with these less studied exposure indices.

### Comparison with other recently published reviews

A review in 2007 ^73^ considered that “ETS exposure may be an important cause of COPD”. However, this conclusion was based on only six studies, one examining absolute risk of COPD in relation to changes in tobacco consumption, and one comparing lung function of employees in bars and restaurants before and after a smoking ban. Also it seemed that at least some of the others considered were not restricted to never smokers.

A review in 2010 ^60^ meta-analysed results from 12 studies and gave an overall estimate of 1.56 (1.40-1.74), somewhat higher than our estimate. Not all of the studies included were of COPD, some being based on chronic bronchitis symptoms. Also, some studies were based on current non-smokers rather than on lifelong never smokers.

In 2013, Bentayeb *et al.* ^61^ reviewed evidence on indoor air pollution and respiratory health in those aged over 65 years. After considering 33 papers (only one ^16^ presenting relevant results on ETS and COPD risk in non-smokers), they reported that the most consistent relationship found was between ETS exposure and COPD risk. However, the findings did not allow causal inference due to heterogeneity of the studies considered, measurement errors in exposure assessment, variable outcome definition, and lack of information on lifetime exposure to air pollution. The authors concluded that more investigations are needed to understand the relationship of indoor air pollution to respiratory health in the elderly.

A review in 2014 ^74^ reached similar conclusions, the authors stating that “second-hand exposure to tobacco smoke has also been shown to be associated with the risk of COPD, although more robust evidence needs to be generated”. These conclusions were derived from only eight studies, some concerned with respiratory symptoms rather than COPD. Also, one study did not restrict any analyses to never smokers.

A review in 2015 ^62^ included only five studies in the meta-analyses. The estimated risk of COPD in ETS-exposed participants was higher than we estimated, being 1.66 (1.38-2.00) for both sexes combined, 1.50 (0.96-2.28) for males and 2.17 (1.48-3.18) for females. However, these estimates were based on, respectively, three, one and one estimates, the authors examining three further studies but not including them in their meta-analysis due to low study quality. However, two of the studies they did include were not based on lifelong never smokers. Many other studies that might have been included were not. The authors noted that “the few existing studies on second-hand smoke exposure and COPD differ considerably, although the results indicate a positive association” and that “further research is needed, to provide more adequate primary studies which account for confounding and other biases”.

A review in 2016 ^63^ of “the effects of smoking on respiratory health” also considered effects of ETS exposure. However, only three studies were cited, two not satisfying our inclusion criteria. Noting the variability in the results, the authors only pointed to the need for additional studies.

Generally these reviews point to an association between ETS exposure and risk of COPD without concluding that a causal relationship has clearly been established. The present review, which includes far more studies, confirms the association and provides evidence that is strongly suggestive of a true effect. While this suggestion is not inconsistent with the view of the Global Burden of Disease Study 2017 ^75^ that second-hand smoke is a risk factor for COPD, limitations of the evidence, discussed above, precludes a more definitive conclusion.

### Another relevant publication

In response to a comment from a reviewer (Dr Maio), we updated our searches by a further two years. While this identified an additional 99 publications, only one ^76^ satisfied our inclusion criteria. That paper reported age and sex adjusted hazard ratio estimates by level of ETS exposure, which, when combined, gave an exposed/unexposed estimate of 2.25 (95% CI 1.05-4.82). Including this estimate, based on only 33 COPD cases, had little effect on the meta-analysis results shown in Table 4. Thus, the overall random-effects estimate of 1.20 (95% CI 1.08-1.34) for all COPD was changed only to 1.22 (1.09-1.36), while that for Asia was changed only from 1.34 (1.08-1.67) to 1.38 (1.11-1.72).

## Conclusion

Taken in conjunction with the strong association of smoking with COPD, the significant relationship seen for the main index of ETS exposure, and the evidence of a dose-response relationship is highly suggestive that ETS also increases risk of COPD. However, the absence of well-designed and fully reported large studies, and the limitations noted above make it difficult to obtain an accurate estimate of the true magnitude of any possible effect. More definitive studies are required to reach a firmer conclusion. 

## Data availability

### Underlying data

There were no underlying data associated with this article

### Extended data

**Supplementary file 1:** Rejected studies: In preparing the tables and figures in this document, certain papers that might be thought to provide relevant data have not been referred to. For each of these papers, this appendix notes the authors, date of publication and country and the reasons for not referring to them. https://doi.org/10.17605/OSF.IO/8APGK ^77^

**Supplementary file 2:** PRISMA checklist

https://doi.org/10.17605/OSF.IO/8APGK ^77^

**Supplementary file 3:** Forest plots in Excel

https://doi.org/10.17605/OSF.IO/8APGK ^77^
