# Supplementary material for: Epidemiological evidence relating environmental smoke to COPD in lifelong non-smokers: a systematic review
Source: F1000Res. 2020 Jan 9;7:146. Originally published 2018 Feb 5. [Version 3] doi: 10.12688/f1000research.13887.3 (PMC6953425; doi:10.12688/f1000research.13887.3)
Supplement: Peer review history

# Peer review: Reviewer response for version 3

**DOI:** 10.5256/f1000research.24188.r58417

**Published:** 2020-01-09

**Authors:** Sara Maio

**Affiliations:** 1 Pulmonary Environmental Epidemiology Unit, CNR Institute of Clinical Physiology, Pisa, Italy

Now it is ok for me.

I confirm that I have read this submission and believe that I have an appropriate level of expertise to confirm that it is of an acceptable scientific standard.

# Response

**Published:** 2020-01-09

**Authors:** Peter Lee

**Affiliations:** P.N.Lee Statistics and Computing Ltd, UK

Thanks very much.

# Peer review: Reviewer response for version 2

**DOI:** 10.5256/f1000research.23987.r57969

**Published:** 2019-12-30

**Authors:** Yousser Mohammad

**Affiliations:** 1 National Center for Research in Chronic Respiratory Diseases and Comorbidities, Tishreen University School of Medicine, Tishreen University, Latakia, Syria

I find it is now definitely acceptable for indexing. I have nothing to add.

I confirm that I have read this submission and believe that I have an appropriate level of expertise to confirm that it is of an acceptable scientific standard.

# Response

**Published:** 2019-12-30

**Authors:** Peter Lee

**Affiliations:** P.N.Lee Statistics and Computing Ltd, UK

I thank Dr Mohammad, and am glad he is now happy with the paper.

# Peer review: Reviewer response for version 2

**DOI:** 10.5256/f1000research.23987.r57968

**Published:** 2019-12-20

**Authors:** Sara Maio

**Affiliations:** 1 Pulmonary Environmental Epidemiology Unit, CNR Institute of Clinical Physiology, Pisa, Italy

I would like to thank you the authors for their replies.

 As regards my comment about the number of aspects and subsets, I agree with the authors that detailed information is essential to get a good insight of the described topic; however, on the other side, this could make text reading less smooth. Maybe, some aspects could be inserted in an online supplement.

 However, I keep thinking that it is not to overlook the results about the dose-response evidence or the findings of the whole sample meta-analysis (OR 1.20); instead, it seems that the main message of the review is in favor of an absence of relationship between ETS and COPD. And this aspect is reinforced from the paragraph "Comparison with other recently published reviews", in which the reviews, reporting a significant association between ETS and COPD, are all discussed in terms of limitations and not to compare your positive results with those of other authors.

 I agree with the authors that it is difficult to define a causal relationship basing on the published evidences; but, on the other side, I think that the possible relationship between ETS and COPD should be better highlighted. The 2017 Global Burden of Disease study clearly reported that second-hand smoke is a risk factor for COPD deaths and DALYs.

I confirm that I have read this submission and believe that I have an appropriate level of expertise to confirm that it is of an acceptable scientific standard, however I have significant reservations, as outlined above.

# Response

**Published:** 2019-12-30

**Authors:** Peter Lee

**Affiliations:** P.N.Lee Statistics and Computing Ltd, UK

I thank the reviewer for the comments.

As regards the comment about numbers of aspects and subsets, we have left the paper as it is. As a reader our strong preference is to have all the material to be considered in one place, without having to go backwards and forwards between the main paper and supplementary files. Readers can always skip reading information they are not interested in if they wish to.

We have however made a number of changes to the main messages put over. As shown in the red-lining these appear at the end of the abstract, near the end of the discussion section, and in the conclusions section. We now make it clear that the overall association with the main ETS exposure index, coupled with the dose-response evidence, and the evidence on smoking and COPD, provides strong evidence of a possible causal relationship, and note that this is consistent with the Global Burden of Disease 2017 statement that ETS is a risk factor. However we make it clear that one cannot go further based on the evidence - one cannot definitively conclude that ETS causes COPD - still less get very accurate estimation of its possible effect.

I hope that these alterations are sufficient to remove the reviewer's reservations.

# Peer review: Reviewer response for version 1

**DOI:** 10.5256/f1000research.15097.r55818

**Published:** 2019-11-22

**Authors:** Yousser Mohammad

**Affiliations:** 1 National Center for Research in Chronic Respiratory Diseases and Comorbidities, Tishreen University School of Medicine, Tishreen University, Latakia, Syria

The review is interesting, because it highlights methodological issues and gaps in surveys assessing the impact of environmental tobacco smoke on COPD incidence. These gaps and inconsistencies in surveys, lead to inadequacy on assessing the impact, and showing non-confirmative results (OR, RR, etc) on the association between ETS exposure and COPD.

 I recommend it for indexing with minor changes.

 However, the article should address three additional paragraphs or remark on:

1. Public health message of awareness, it should address that, even if non association is found between ETS and COPD, it is still that ETS kills 90,0000 people per year and it is a global public health issue. Haahtela et al. Helsinki by nature. The nature step for respiratory health,Clinical and Transitional Allergy ^1^.
2. Should mention water pipe because it is wide spreading: Yousser Mohammad, Rafea Shaaban, Bassam Abou Al-Zahab, Nikolai Khaltaev, Jean Bousquet, Basim Dubaybo. Impact of active and passive smoking as risk factors for asthma and COPD in women presenting to primary care in Syria: first report by the WHO-GARD survey group ^2^. **WHO **Advisory note 2015: waterpipe tobacco smoking: health effects, research needs and recommended actions by regulators – 2nd ed. World Health Organization. II.WHO Study Group on Tobacco Product Regulation.  ISBN 978 92 4 150846 9 (NLM classification: QV 137) ^3^.
3. Should discuss the difference in the quality of ETS

Not only nicotine but how the smoke is modified in the air and becoming may be more harmful to small airways:

- Lower ignition temperature
- Particles become smaller
- Higher proportion of CO, VOC

Ref found in the article: Mohammad Y. Passive smoking interference with wheezing and asthma Short Review of current knowledge ^4^.

 Wirth et al. Respiratory diseases related to passive smoking ^5^. 

 Beside that, in one study ^6^ the duration of exposure is 2 weeks, maybe we should omit it.

I confirm that I have read this submission and believe that I have an appropriate level of expertise to confirm that it is of an acceptable scientific standard.

## References

[1] : Helsinki by nature: The Nature Step to Respiratory Health. *Clin Transl Allergy*.2019;9: 10.1186/s13601-019-0295-2 57 10.1186/s13601-019-0295-2 31695865PMC6822361

[2] : Impact of active and passive smoking as risk factors for asthma and COPD in women presenting to primary care in Syria: first report by the WHO-GARD survey group. *Int J Chron Obstruct Pulmon Dis*.2013;8: 10.2147/COPD.S50551 473-82 10.2147/COPD.S50551 24124359PMC3794890

[3] : Advisory note: waterpipe tobacco smoking: health effects, research needs and recommended actions for regulators - 2nd edition.2015; Reference source 10.1186/1477-5751-5-17PMC166458317112380

[4] : Passive smoking interference with wheezing and asthma: short review of current knowledge. *Pulmonology and Respiratory Research*.2015;3(1) : 10.7243/2053-6739-3-2 10.7243/2053-6739-3-2

[5] : Passive smoking exposure in adults and chronic respiratory symptoms (SAPALDIA Study). Swiss Study on Air Pollution and Lung Diseases in Adults, SAPALDIA Team. *Am J Respir Crit Care Med*.1994;150(5 Pt 1) : 10.1164/ajrccm.150.5.7952544 1222-8 10.1164/ajrccm.150.5.7952544 7952544

[6] : COPD in never smokers: results from the population-based burden of obstructive lung disease study. *Chest*.2011;139(4) : 10.1378/chest.10-1253 752-763 10.1378/chest.10-1253 20884729PMC3168866

# Response

**Published:** 2019-12-13

**Authors:** Peter Lee

**Affiliations:** P.N.Lee Statistics and Computing Ltd, UK

**Reply to Yousser Mohammad**

Dr Mohammad suggests that I should cite a statement by WHO that ETS is responsible for about 900,000 deaths per year from all causes combined. We would rather not do this for two reasons. First, the paper is specifically about ETS and COPD, so does not need to stray into the relationship between ETS and other causes of death. Secondly, estimation of the effect of ETS on overall mortality is extremely complex and citation of a single estimate is questionable. We have in fact published widely on the evidence relating ETS to other diseases, such as lung cancer (Lee *et al*., 2016a; Lee *et al*., 2002), other cancers (Lee, 2002; Lee and Hamling, 2006; Lee and Hamling, 2016; Lee *et al*., 2016b), stroke (Lee and Forey, 2006; Lee *et al*., 2017b), heart disease (Lee *et al*., 2017a), and asthma (Lee and Forey, 2007), and find little evidence of an effect as large as WHO claims. For lung cancer, for example, we concluded (Lee *et al*., 2017b) that any causal relationship is not convincingly demonstrated, as most, if not all, of the relationship with ETS can be explained by confounding adjustment and misclassification correction.

Dr Mohammad also suggests that I mention waterpipes. We would prefer not to do this as the paper is about ETS exposure from conventional cigarettes and as we have not formally reviewed the evidence relating to waterpipes. We have made it clearer in the introduction that the paper concerns ETS from cigarettes.

He also suggests that we should discuss differences in the quality of ETS. We assume that he is pointing out that ETS from various products may not have the same composition or effects as from conventional cigarettes. But we are not concerned with ETS from products other than cigarettes.

He suggests that we refer to evidence relating ETS to wheezing and asthma. While the paper he cites does not actually mention COPD, we do now include a statement in the text at the end of paragraph 2 of the discussion.

**References**

Lee, P.N., 2002. Environmental tobacco smoke and cancer of sites other than the lung in adult non-smokers. Food Chem. Toxicol. 40, 747-766.

Lee, P.N., Forey, B.A., 2006. Environmental tobacco smoke exposure and risk of stroke in nonsmokers: a review with meta-analysis. J. Stroke Cerebrovasc. Dis. 15, 5, 190-201.

Lee, P.N., Forey, B.A., 2007. The role of environmental tobacco smoke in asthma induction and exacerbation in children and adults. Nova Science Publishers, Inc., New York.

Lee, P.N., Forey, B.A., Hamling, J.S., Thornton, A.J., 2017a. Environmental tobacco smoke exposure and heart disease: A systematic review. World J. Metaanal. 5, 2, 14-40. DOI:10.13105/wjma.v5.i2.14.

Lee, P.N., Fry, J.S., Forey, B., Hamling, J.S., Thornton, A.J., 2016a. Environmental tobacco smoke exposure and lung cancer: a systematic review. World J. Metaanal. 4, 2, 10-43. DOI:10.13105/wjma.v4.i2.10.

Lee, P.N., Fry, J.S., Forey, B.A., 2002. Revisiting the association between environmental tobacco smoke exposure and lung cancer risk. V. Overall conclusions. Indoor Built Environ. 11, 59-82. DOI:10.1177/1420326X0201100202.

Lee, P.N., Hamling, J., 2006. Environmental tobacco smoke exposure and risk of breast cancer in nonsmoking women: a review with meta-analyses. Inhal. Toxicol. 18, 14, 1053-1070.

Lee, P.N., Hamling, J.S., 2016. Environmental tobacco smoke exposure and risk of breast cancer in nonsmoking women. An updated review and meta-analysis. Inhal. Toxicol. 28, 10, 431-54. DOI:10.1080/08958378.2016.1210701.

Lee, P.N., Thornton, A.J., Forey, B.A., Hamling, J.S., 2017b. Environmental tobacco smoke exposure and risk of stroke in never smokers: An updated review with meta-analysis. J. Stroke Cerebrovasc. Dis. 26, 1, 204-216. DOI:10.1016/j.jstrokecerebrovasdis.2016.09.011.

Lee, P.N., Thornton, A.J., Hamling, J.S., 2016b. Epidemiological evidence on environmental tobacco smoke and cancers other than lung or breast. Regul. Toxicol. Pharmacol. 80, 134-163. DOI:10.1016/j.yrtph.2016.06.012.

# Peer review: Reviewer response for version 1

**DOI:** 10.5256/f1000research.15097.r33219

**Published:** 2018-05-11

**Authors:** Sara Maio

**Affiliations:** 1 Pulmonary Environmental Epidemiology Unit, CNR Institute of Clinical Physiology, Pisa, Italy

The review could be of interest but some some major revisions are needed.

 In particular, it would be important to update the papers selected for the revision because many other scientific evidences were published after June 2016.

 Moreover, too many aspects and subsets are taken into account; they did not add information and they make the paper hard to read and the message not clear.

 As regards to the subsets chosen for the meta-analysis, it would be important to take into account the publication period before and after the smoking ban, because it determined a variation in the exposure of the people to passive smoke and the related effects.

 In the discussion, section "publication bias", it was reported that " One must note, though, that various large longitudinal studies, e.g. 66–69, reported results relating ETS to smokingrelated diseases such as lung cancer or heart disease, but did not do so for COPD. If any relationship had been seen, these studies might well have been reported.".

 The reviewer thinks that this sentence is too strict and not supported by evidences.

 Overall the discussion seems to set up versus a negative approach against the results of the other published reviews; on the other side, it did not discuss the results regarding the negative effect of exposure to ETS found using meta-analyses. The reviewer thinks that it is not to overlook the results about the dose-response evidence or the findings of the whole sample meta-analysis (OR 1.20) etc...   

I confirm that I have read this submission and believe that I have an appropriate level of expertise to confirm that it is of an acceptable scientific standard, however I have significant reservations, as outlined above.

# Response

**Published:** 2018-05-15

**Authors:** Peter Lee

**Affiliations:** P.N.Lee Statistics and Computing Ltd, UK

Dear Dr Maio,

 I thank you for your comments which I respond to below on behalf of my co-authors.  The text of the paper has not yet been altered as the editorial team advise that I wait for the additional referee reports before doing so.

Updating selected papers  You say that "many other scientific evidences were published after June 2016.  However, we have updated our searches to cover more recent papers, and only found one paper which satisfied the inclusion criteria. This was a report by Ukawa et al in 2017 (International Journal of Public Health, vol 62, pp 489-494) which presented results for at home passive smoking exposure from the Japan Collaborative cohort study, based only on 33 cases. Rather than updating the whole range of analysis results, we intend simply to refer to this additional study, and the effect it had on the overall effect estimate, in a comment at the end of the discussion section.  If you think there are other important papers we have missed please let us know what they are.

Too many aspects and subsets are taken into account  We have published a number of previous reviews of the relationship of passive smoking to other diseases, and this style has never before been criticized.  In our view it is important to fully describe how the association of interest varies by the source of exposure and by study characteristics, and also by the definition of disease.  One cannot get a good insight without these details.

Taking into account the publication period before and after the smoking ban  You say "the smoking ban" but there are many smoking bans, different in type and different in timing. In the US for example different states, and different locations within states, brought in bans at different times. When one also considers the long latent period of COPD, with deaths post-ban perhaps due to exposures pre-ban, and the fact that in some studies some COPD cases occur pre-ban and some post-ban, we did not consider it useful to attempt the required analysis.

Publication bias   We made the point (also made in other passive smoking reviews) that some large cohort studies are known to have published positive relationships relating passive smoking to other diseases when they did not publish results relating passive smoking to COPD.  Surely it is quite likely that they did not find a.positive relationship for COPD?   In my view large cohort studies ought to publish passive smoking results for all diseases with sufficient cases, but often they do not.  Our comment is supported by the evidence as to what has and has not been published - though this does not prove that all such studies found no positive association with COPD, the likelihood is there.  The argument is similar to the general one for publication bias.  We don't generally have evidence that  papers showing no association are less likely to be submitted or accepted than papers finding an association, but it is highly plausible.

Negative approach against other reviews   We state the reasons why these other reviews are limited.

Overlooking evidence suggesting an association, such as the dose-response results and the overall meta-analysis results   In paragraph 2 of the discussion we refer to the overall meta-analysis results and the dose-response results, and then go on to discuss why these results are only suggestive of a causal relationship.  The overall association of 1.20 with passive smoking at home, though highly statistically significant, is quite small in magnitude, and it is certainly possible that it may be explicable in terms of bias.  In our review of passive smoking and lung cancer (World Journal of Meta-Analysis, 2017, 4, 10-43) we were able to demonstrate quite clearly that a similar sized association could plausibly be explained by a combination of uncontrolled confounding and misclassification of smoking status.  Though the data for COPD are not extensive enough to readily allow such adjustments, we would be extremely nervous in saying that the association is more than suggestive of a causal relationship.  Nevertheless, we will look again at the wording we have used and try to make our argument clearer.

Conclusions drawn not adequately supported by the results presented   This really relates to the previous point.  We believe our conclusions are supported by the results.

 We would be happy to hear your reactions to our replies.

 Yours sincerely

 Peter Lee (and co-authors)
